# MiR-92a Family: A Novel Diagnostic Biomarker and Potential Therapeutic Target in Human Cancers

**DOI:** 10.3389/fmolb.2019.00098

**Published:** 2019-10-01

**Authors:** Min Jiang, Xuelian Li, Xiaowei Quan, Xiaoying Li, Baosen Zhou

**Affiliations:** Department of Epidemiology, School of Public Health, China Medical University, Shenyang, China

**Keywords:** miR-92a, RGS3, neoplasms, diagnosis, prognosis

## Abstract

**Purpose:** This study tried to explore whether members of miR-92a family contribute to early diagnosis and prognosis for human cancers and how they work.

**Methods:** Integrated meta-analysis retrieved from public repositories was employed to assess the clinical roles of the miR-92a family for cancer diagnosis and prognosis. Expression level of miR-92a was detected by the TCGA database and was confirmed by non-small-cell lung cancer (NSCLC) tissues. Targets of miR-92a were predicted using starbase, and validated by dual luciferase assay. Correlation between miR-92a and the target gene was assessed by linkedOmics while expression of the target gene and its role in cancer prognosis were analyzed with UALCAN and Gepia.

**Results:** We recognized the miR-92a family could serve as a potential diagnostic biomarker with a pooled sensitivity of 0.85 [0.81–0.88] and specificity of 0.86 [0.83–0.90]. The overall hazard ratio (HR) was 2.26 [95% CI: 1.70–3.00] for high expression groups compared to low expression groups. Expression of miR-92a was identified to be upregulated in NSCLC, especially in lung squamous cell carcinoma (LUSC). Results from starbase and dual luciferase assay indicated the regulator of G-protein signaling 3 (RGS3) was a direct target of miR-92a. Statistical negative correlation was found for the expression of miR-92a and RGS3. In addition, expression of RGS3 was downregulated in NSCLC and patients with the high expression had a poor prognosis (HR = 1.3) for LUSC patients. However, results were to the contrary for lung adenocarcinoma (HR = 0.7).

**Conclusion:** This study revealed that miR-92a family could be ideal biomarkers for cancer diagnosis and prognosis, which might function through targeting RGS3.

## Introduction

MicroRNAs (miRNAs) are small, conserved, single-stranded, non-coding RNAs, which adjust gene expression in the post-transcriptional level (Lagos-Quintana et al., 2001). MiRNAs participate in a series of processes including cellular proliferation, differentiation, and apoptosis and promote tumorigenesis and metastasis (Nana-Sinkam and Croce, 2011; Bracken et al., 2016). The MiR-92a family, including miR-25, miR-363, and miR-92a, arise from three homologous clusters, namely, miR-106b-25, miR-106a-363, and miR-17-92 (Olive et al., 2010). MiR-25 is situated in the thirteenth intron of minichromosome maintenance protein 7 (MCM7) gene in 7q22.1 of human chromosome, while miR-92a-1 is located within the third intron of the chromosome 13 open reading frame 25 (C13orf25) gene. Both miR-363 and miR-92a-2 whose pri-miRNAs have been proven to be Kis2 ncRNAs are encoded in the miR-106-363 cluster from q26.2 of X chromosome. What's more, miR-92a-1 and miR-92a-2 would be processed into mature miR-92a. Studies indicate that miR-92a family members are involved not only in the formation of blood vessels but also in the development of some mammalian organs (Ventura et al., 2008). Moreover, the abnormal expression of miR-92a members has been found in different malignant human tumors (Hu et al., 2012; Zhu et al., 2014; Fan et al., 2016; Motawi et al., 2016; Elhamamsy et al., 2017; Fujiwara et al., 2017; Ding et al., 2018).

The ectopic expression for this family might promote tumor proliferation, metastasis, invasion while inhibit tumor apoptosis. Several studies have indicated that miR-25 regulated the G1/S and G2/M cell cycle arrest by directly targeting p57, cyclin E2, CDK2, CDC42, and EZH2 to promote cell proliferation (Kim et al., 2009; Esposito et al., 2012; Zhao et al., 2014; Yang et al., 2015). Interestingly, the ectopic expression of miR-25 might also promote invasion and migration of cancer cells through KLF4-ERK and RhoGDI1-WNT/β-catenin signaling pathways (Wang et al., 2015; Ding et al., 2018). Upregulation of miR-92a might lead to an accumulation of the G1 phase stem cells and a reduction for S phase cells in colorectal cancer (Xu et al., 2018). In addition, it might promote colorectal cancer cell growth and migration by inhibiting the expression of KLF4 (Lv et al., 2016). This inhibition of proliferation also occurred in cervical cancer and osteosarcoma via targeting p21 and FBXW7, respectively (Jiang et al., 2017; Su et al., 2017). Moreover, miR-92a could regulate oral squamous cell carcinoma cell growth by targeting FOXP1 expression (Guo et al., 2018). The overexpression of miR-92a was proven to induce the EMT process through regulating PI3K/AKT signaling activities via directly targeting PTEN, therefore promoting NSCLC cell migration and invasion as well as tumor growth (Lu C. et al., 2017). Regarding the involvement of miR-363 in cancer development and progression, studies have shown that miR-363 promoted tumor cell proliferation, invasion, and metastasis by regulating not only SP1 and Notch1, but the PIK3CA-PI3K/AKT pathway (Song et al., 2015; Liu et al., 2017; Ying et al., 2017). Downregulated miR-363 could enhance the expression level of SOX4 and lead to the process of EMT and metastasis of colorectal cancer (Hu et al., 2016).

Given that these various observations have been made in multiple publications, there is a strong need to assess the potential value of the miR-92a family for human cancers. Therefore, we performed this research to evaluate the performance of the miR-92a family in early diagnosis and precise prognosis prediction, as well as its specific mechanism, in human cancers.

## Materials and Methods

### Integrated Analysis of miR-92a Family for Cancer Diagnosis and Prognosis

We searched the PubMed and Embase databases to retrieve all relevant articles based on the Preferred Reporting Items for Systematic Reviews and Meta-Analyses (PRISMA). The search strategy was (hsa-miR-92a or miR-92a or microRNA-92a or miR92a or hsa-miR-25 or miR-25 or microRNA-25 or miR25 or hsa-miR-363 or miR-363 or microRNA-363 or miR363) and (tumor or carcinoma or neoplasm or cancer), which were updated until March 10, 2018. The reference list was also retrieved. Criteria were drafted for literature screening. Demographic information and data for meta-analysis including sensitivity (SEN), specificity (SPE), true positive (TP), false positive (FP), false negative (FN), true negative (TN), HR with 95% CI was collected from included literature by two authors (MJ and XY-L) independently. The quality of publications with diagnostic data was evaluated based on the Quality Assessment of Diagnostic Accuracy Studies 2 guidelines (QUADAS-2), while guidelines from the Newcastle-Ottawa Scale (NOS) were followed for quality assessment of publications with prognostic data (Stang, 2010; Whiting et al., 2011).

### TCGA Datasets and Bioinformatics Websites

A RNA-seq dataset of mRNA and miRNA expression including 1,129 samples (515 lung adenocarcinoma (LUAD) tissues, 503 LUSC tissues and 111 normal tissue samples) downloaded from TCGA was used to evaluate the expression level of miR-92a and the expression of target gene by UALCAN (http://ualcan.path.uab.edu) (Chandrashekar et al., 2017). Since there was no single expression data for miR-92a, we analyzed the expression level of MIR17HG, a host gene of miR-92a. In addition, LinkedOmics (http://www.linkedomics.org) (Vasaikar et al., 2018) was employed to analyze the correlation between the expression of miR-92a and the target gene by enrolling a total of 789 samples (447 LUAD tissues and 342 LUSC tissues). For the survival analysis, 959 tumor tissue samples were enrolled, including 477 LUAD tissues and 482 LUSC tissues, which were analyzed by Gepia (http://gepia2.cancer-pku.cn) (Tang et al., 2019).

### Patients and Samples

Non-small-cell lung cancer (NSCLC) tissues with adjacent normal tissues were obtained from 52 patients with NSCLC between July 2010 and December 2014. All tissues were quick-frozen in liquid nitrogen and stored at −80°C. All patients provided the informed consent. This research was approved by the Institutional Review Board of China Medical University.

### Cell Line and Cell Culture

The human NSCLC cell line A549 was purchased from GeneChem (Shanghai, China) and was cultured in Roswell Park Memorial Institute (RPMI) 1640 medium with 10% fetal bovine serum (FBS) at 37°C with 5% CO_2_.

### Cell Transfection

For overexpression of miR-92a, the cells were transiently transfected with 100 nM miR-92a mimics and negative controls synthesized by JTS (Wuhan, China) using jetPRIME (Polyplus-transfection, France). For overexpression of RGS3, 1 μg RGS3 vector and the negative control (JTS, Wuhan, China) were transfected using jetPRIME as described above.

### RNA Isolation and qRT-PCR Analysis

Total RNA was exacted from tissue samples using RNAiso Plus (Takara Bio Inc., Japan) following the manufacture's procedure. To detect the RNA expression levels of miR-92a, TaqMan MicroRNA Reverse Transcription Kits (Applied Biosystems, USA) were used for cDNA synthesis and TaqMan MicroRNA Assays (Applied Biosystems, USA) were used for quantitative PCR. U6 was used as an internal control. The PCR was repeated three times for every sample. The relative expression was calculated by the 2^−ΔΔCt^ method.

### Identification of Target mRNA of miR-92a

Starbase v2.0 (Li et al., 2014) was utilized to predict the potential target mRNA of miR-92a. Starbase showed the results from five informatics databases including TargetScan, PicTar, RNA22, PITA and miRanda/mirSVR. The potential mRNAs with all these five databases were screened out, which indicated a potential target site.

### Luciferase Reporter Assay

Luciferase reporters were generated by JTS (Wuhan, China). The potential binding sites in 3′-UTR of RGS3 were inserted into the Dual-Luciferase vector. A vector containing the mutant 3′-UTR fragment of RGS3 was constructed as a negative control. The luciferase reporters were co-transfected with miR-92a mimics or negative control by jetPRIME (Polyplus-transfection, France). At 48 h after transfection, the luciferase activities were detected with the Dual-Luciferase Reporter Assay System (Promega) in Synergy H1 system (Bio Tek).

### Statistical Analysis

Overall diagnostic estimates including SPE, SEN, negative likelihood ratio (NLR), positive likelihood ratio (PLR) and the diagnostic odd ratio (DOR) were assessed by the random effect regression model. Summary receiver operating characteristic (SROC) and value of area under the SROC curve (AUC) were constructed and evaluated. A detailed diagnostic analysis was conducted including meta-regression analyses and subgroup analyses (grouped according to specimen, ethnicity, miRNA profiling, types of control, cancer-type, and stage) to identify and decrease the heterogeneity. Moreover, the pooled HR with its 95% CI was evaluated for prognostic meta-analysis. Simultaneously, heterogeneity among these publications was analyzed by *I*^2^ value and *Q*-test (Higgins et al., 2003). *P*-value of *Q*-test <0.05 or *I*^2^ ≥ 50% indicated a significant heterogeneity existed. Deek's funnel plot asymmetry test was performed to judge the possibility of publication bias for diagnostic meta-analysis while Begg's and Egger's tests for prognostic meta-analyses. All integrated analysis were conducted by STATA 11.0 (STATA-Corp, College Station, TX, version 11.0). RevMan 5.3 software (version 1.4) was employed to assess the quality of enrolled literature for diagnostic meta-analysis. While all other statistical analyses were conducted using SPSS 20.0 (IBM, NY). The *t*-test was performed to compare the means of two groups. Analysis of variance was conducted to compare the results among three or more groups. Dunnett-*t*-test was employed to compare the results between different experiment groups with a same control group. Spearman correlation analysis was used to assess the correlation between expression level of miR-92a and the target gene. A value of *P* < 0.05 was defined as indicating statistical significance with two-tailed.

## Results

### Diagnostic Meta-Analyses

**Figure 1:**
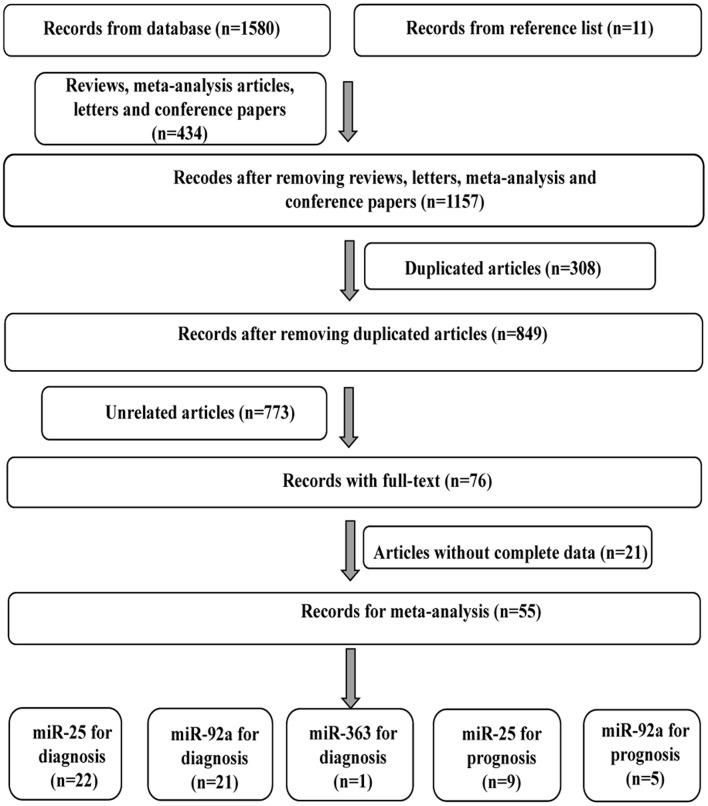
Flow diagram of study selection for cancer diagnostic and prognostic meta-analysis of miR-92a family.

**Figure 2:**
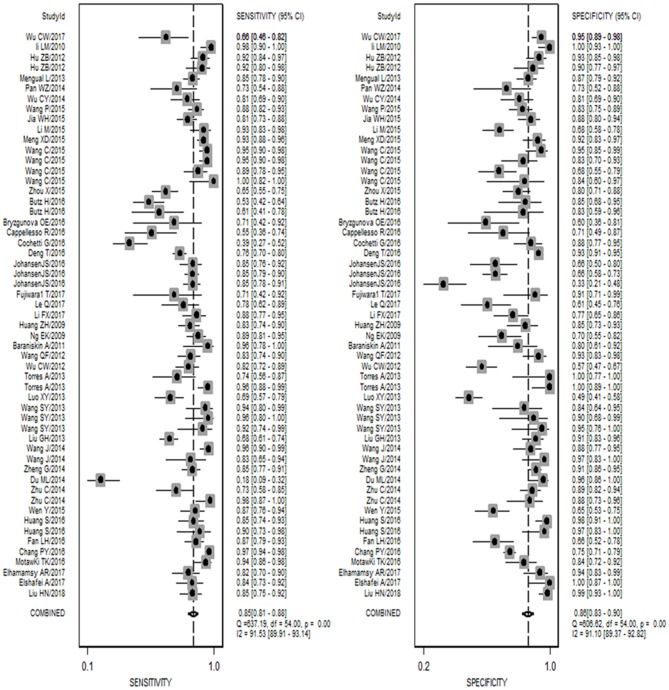
Forest plots of sensitivity and specificity for the cancer diagnosis of miR-92a family. Both the sensitivity and specificity of each study were showed by each square with its 95% confidence interval showed by the error bars.

As shown in Figure 1, 42 publications with 55 researches, including 4,526 cases and 4,304 controls, were analyzed for detailed information (Table S1, Figures S1, S2). For overall diagnostic performance, results of the random-effect model demonstrated overall SEN and SPE were 0.85 [0.81–0.88] and 0.86 [0.83–0.90], respectively (Figure 2). The value of AUC was 0.92 [95% CI: 0.90–0.94] (Figure S3). Results for meta-regression analyses indicated miRNA profiling (single or multiple) might explain the heterogeneity in SEN (P < 0.05) as shown in Figure S4. A *P*-value equal to 0.92 for Deek's funnel plot asymmetry test indicated that there was no publication bias among these researches (Figure S5).

As shown in Table 1, the SEN and SPE for miR-25 were 0.84 [95% CI: 0.79–0.89] and 0.82 [95% CI: 0.77–0.87], respectively (Figure S6), while AUC was 0.90 [0.87–0.93] (Figure S7). For lung cancer, miR-25 indicated a significantly high diagnostic value with SEN: 0.93 [95% CI: 0.88–0.96] and SPE: 0.84 [95% CI: 0.75-0.91].

**Table 1:** The overall and subgroup diagnostic meta-analysis.

**Subgroups**	**No. of studies**	**SEN [95% CI]**	**SPE [95% CI]**	**PLR [95% CI]**	**NLR [95% CI])**	**DOR [95% CI])**	**AUC [95% CI]**
Overall	55	0.85 [0.81–0.88]	0.86 [0.83–0.90]	6.3 [4.8–8.2]	0.17 [0.13–0.22]	37 [24–56]	0.92 [0.90–0.94]
MiR-25	30	0.84 [0.79–0.89]	0.82 [0.77–0.87]	4.8 [3.6–6.4]	0.19 [0.14–0.26]	25 [15–44]	0.90 [0.87–0.93]
**Specimen**							
Plasma	7	0.84 [0.74–0.91]	0.78 [0.70–0.84]	3.8 [2.8–5.1]	0.20 [0.12–0.33]	19 [10–36]	0.87 [0.84–0.90]
Serum	18	0.88 [0.82–0.92]	0.85 [0.77–0.91]	5.9 [3.7–9.3]	0.15 [0.09–0.22]	40 [19–88]	0.93 [0.90–0.95]
Urinary	4	0.68 [0.52–0.81]	0.81 [0.73–0.88]	3.6 [2.1–6.2]	0.39 [0.23–0.66]	9 [3–25]	0.83 [0.80–0.86]
**Ethnicity**							
Asian	18	0.86 [0.81–0.90]	0.84 [0.78–0.89]	5.5 [3.9–7.7]	0.16 [0.11–0.23]	34 [18–62]	0.92 [0.89–0.94]
Caucasian	12	0.80 [0.69–0.89]	0.79 [0.68–0.87]	3.9 [2.4–6.2]	0.25 [0.14–0.42]	16 [6–38]	0.87 [0.83–0.89]
**Control-type**							
HC	22	0.86 [0.80–0.90]	0.82 [0.75–0.87]	4.8 [3.3–6.8]	0.17 [0.12–0.25]	27 [14–53]	0.91 [0.88–0.93]
BD	5	0.81 [0.56–0.94]	0.80 [0.65–0.89]	4.0 [2.1–7.6]	0.24 [0.09–0.65]	17 [4–71]	0.87 [0.83–0.89]
**Cancer-type**							
Lung cancer	5	0.93 [0.88–0.96]	0.84 [0.75–0.91]	6.0 [3.5–10.2]	0.09 [0.05–0.15]	69 [25–189]	0.95 [0.93–0.97]
Digestive Neoplasms	13	0.84 [0.78–0.88]	0.79 [0.67–0.87]	4.0 [2.4–6.6]	0.20 [0.14–0.30]	20 [9–44]	0.89 [0.85–0.91]
Urogenital Neoplasms	9	0.78 [0.64–0.88]	0.87 [0.84–0.90]	6.2 [4.3–9.0]	0.25 [0.14–0.44]	25 [10–62]	0.90 [0.87–0.92]
**MiRNA-profiling**							
Single	15	0.76 [0.66–0.83]	0.82 [0.74–0.88]	4.2 [3.0–5.8]	0.30 [0.21–0.41]	14 [9–23]	0.86 [0.83–0.89]
Multiple	20	0.88 [0.82–0.92]	0.84 [0.77–0.89]	5.6 [3.7–8.3]	0.14 [0.10–0.22]	39 [19–79]	0.93 [0.90–0.95]
MiR-92	27	0.87 [0.82–0.91]	0.90 [0.84–0.93]	8.5 [5.5–13.2]	0.15 [0.10–0.21]	58 [32–107]	0.94 [0.92–0.96]
**Specimen**							
Plasma	12	0.84 [0.71–0.91]	0.85 [0.76–0.91]	5.6 [3.4–9.1]	0.19 [0.11–0.35]	29 [13–63]	0.91 [0.88–0.93]
Serum	9	0.86 [0.80–0.90]	0.95 [0.88–0.98]	16.0 [6.8–37.9]	0.15 [0.11–0.22]	105 [40–278]	0.94 [0.92–0.96]
Tissue	4	0.95 [0.90–0.97]	0.94 [0.83–0.98]	14.8 [5.2–42.2]	0.06 [0.03–0.11]	266 [68–1041]	0.96 [0.94–0.98]
**Ethnicity**							
Asian	20	0.87 [0.80–0.91]	0.89 [0.83–0.93]	7.7 [5.0–11.8]	0.15 [0.10–0.23]	52 [28–94]	0.94 [0.92–0.96]
Caucasian	4	0.87 [0.69–0.95]	0.94 [0.42–1.00]	13.6 [0.8–239.2]	0.14 [0.05–0.40]	95 [3–3301]	0.94 [0.91–0.96]
**Control-type**							
HC	18	0.86 [0.78–0.91]	0.91 [0.85–0.95]	9.7 [5.7–16.7]	0.16 [0.10–0.24]	62 [32–120]	0.95 [0.92–0.96]
BD	7	0.92 [0.83–0.96]	0.84 [0.68–0.93]	5.7 [2.6–12.7]	0.10 [0.04–0.23]	57 [12–269]	0.95 [0.92–0.96]
**Cancer-type**							
Colorectal cancer	16	0.85 [0.77–0.91]	0.89 [0.81–0.94]	7.8 [4.5–13.6]	0.16 [0.10–0.27]	47 [21–105]	0.94 [0.91–0.95]
Gastric cancer	4	0.88 [0.75–0.95]	0.93 [0.86–0.97]	13.0 [6.0–28.0]	0.13 [0.06–0.28]	100 [31–329]	0.96 [0.94–0.98]
**MiRNA-profiling**							
Single	16	0.81 [0.70–0.88]	0.81 [0.73–0.87]	4.3 [3.0–6.2]	0.24 [0.16–0.37]	18 [10–34]	0.88 [0.85–0.90]
Multiple	20	0.89 [0.84–0.92]	0.91 [0.85–0.94]	9.7 [6.1–15.5]	0.12 [0.09–0.17]	78 [43–144]	0.95 [0.93–0.97]

*AUC, area under the curve; BD, benign pulmonary disease; DOR, Diagnostic Odds Ratio; HC, healthy control; NLR, negative likelihood ratio; No. of studies, the number of the studies; PLR, positive likelihood ratio; SEN, sensitivity; SPE, specificity*.

The forest plot described the diagnostic effectiveness of miR-92a in human cancers with a diagnostic value of 0.87 [95% CI: 0.82–0.91] for SEN and 0.90 [95% CI: 0.84–0.93] for SPE, respectively (Figure S8). A value of 0.94 (95% CI: 0.92–0.96) for AUC was obtained from the result of SROC (Figure S9). Results of the subgroup analysis indicated studies using tissue samples exhibited highest diagnostic accuracy compared to studies using plasma and serum samples. In gastric cancer, miR-92a showed a significantly high diagnostic value of 0.93 [95% CI: 0.88–0.96] for SEN and 0.84 [95% CI: 0.75–0.91] for SPE, respectively.

### Prognostic Meta-Analyses

Fourteen publications with 16 studies were enrolled for the pooled prognostic meta-analysis, main characteristics of which were displayed in Tables S2, S3. Primary sample types included tissue and serum. Types of cancer included colorectal, gastric, breast, lung and hepatocellular cancer, non-muscle invasive bladder cancer, esophageal squamous cell carcinoma, and epithelial ovarian cancer. The expression level of the miR-92a family was tested by the qRT-PCR. U6 and miR-16 were the most common reference miRNAs. The major outcomes consisted of the overall survival (OS), relapse free survival (RFS), disease free survival (DFS), progression-free survival (PFS), and metastasis free survival (MFS). Quality of these included researches was generally good according to the guidelines of NOS.

**Figure 3:**
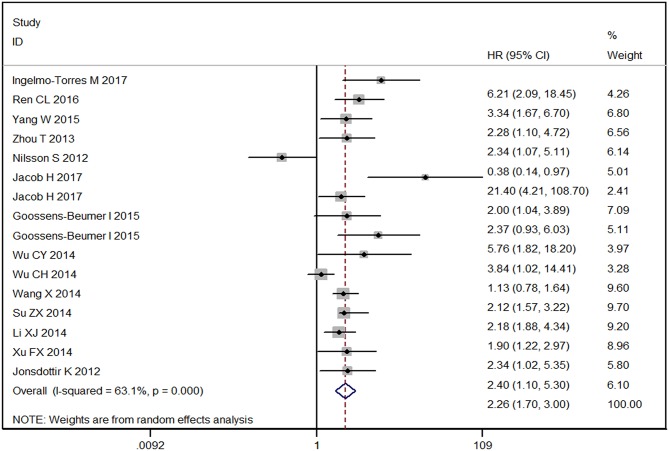
Forest plot of the prognostic meta-analysis of the association between miR-92a family and the risk of cancers. HR, hazard radio; 95% CI, 95% confidence interval.

As shown in Figure 3, the overall HR was 2.26 [1.70–3.00] with *P* < 0.001 for high vs. low expression level of miR-92a family. No obvious publication bias was found since *P-*value for Egger's regression intercept was 0.958 (Figure S10). The forest plot described the prognostic role of miR-25 for human cancers with an overall corrected HR of 2.22 [95% CI: 1.67–2.97] as shown in Figure S11. The statistical difference was significant with Z of 5.42 and *P* < 0.001, indicating that high-miR-25-expression groups had a higher risk of death probability or disease progression than the low-expression groups. The overall HR was 2.11 [95% CI: 0.96–4.65] with Z = 1.85 and *P* = 0.064 for high expression groups of miR-92a vs. low expression groups as described in Figure S12, which meant that the low and high miRNA-expression groups had the same risk of disease progression or death possibility.

### miR-92a Is Upregulated in NSCLC

**Figure 4:**
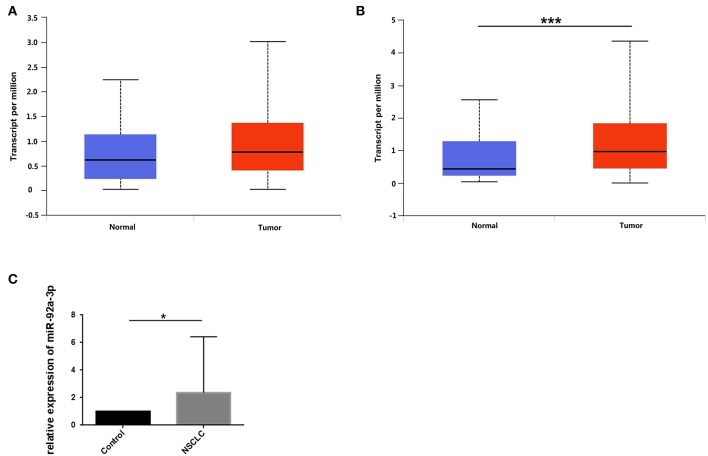
miR-92a is upregulated in NSCLC. **(A,B)** Expression of miR-92a in lung adenocarcinoma **(A)** and lung squamous cell carcinoma **(B)**, values are expressed as Transcript per million, ***p <.001, *t*-test; **(C)** Expression of miR-92a in non-small-cell lung cancer tissues, normalized to the mean of control group and expressed as 2–ΔΔCt, *p < 0.05, *t*-test.

To screen out the expression level of miR-92a in NSCLC, we downloaded the expression of miR-92a in NSCLC from TCGA and further detected the expression in tumor samples by qRT-PCR. As results showed that the high expression of miR-92a in LUSC was found from the TCGA dataset (*P* > 0.05, Figure 4A; *P* < 0.001, Figure 4B) and the 52 pairs of NSCLC and adjacent normal samples (*P* = 0.019, Figure 4C).

### RGS3 Is Targeted by miR-92a in NSCLC

**Figure 5:**
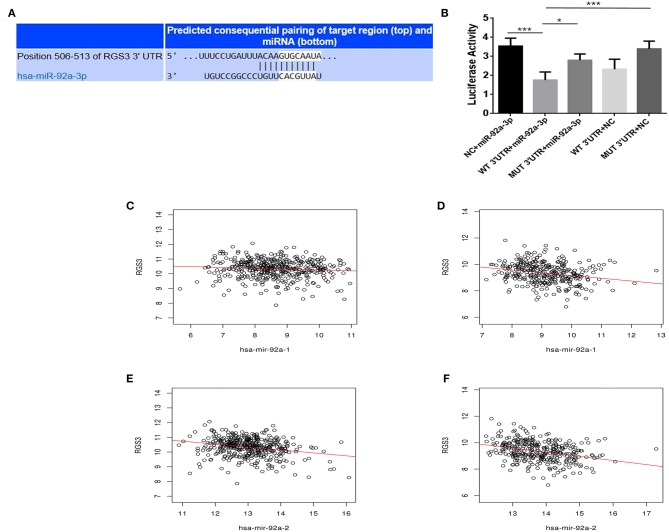
RGS3 is targeted by miR-92a. **(A)** Putative binding sites in the RGS3 3'UTR for miR-92a by starbase. **(B)** miR-92a targets the RGS3 3'-UTR. Luciferase activity of the RGS3 reporter with a wild or mutated miR-92a binding site was measured at 48 h post-transcription with miR-92a mimics or negative control, *p < 0.05, ***p < 0.001, Dunnett-*t*-test. **(C–F)** Expression of miR-92a was negatively correlated with the level of RGS3 in NSCLC from TCGA, r value and *p*-value were determined using spearman correlation analysis.

To investigate the mechanisms of miR-92a, the target genes of miR-92a were predicted in the starbase, and RGS3 was predicted as a target of miR-92a (Figure 5A). The dual-luciferase reporter assay further verified that luciferase expression was inhibited by co-transfection with the wild-3'UTR of RGS3 and miR-92a mimic, which indicated that RGS3 was a target gene of miR-92a (Figure 5B). Then we examined the association of RGS3 and miR-92a expression in the TCGA NSCLC cohort, demonstrating a significant negative correlation (for miR-92a-1, *r* = −0.093, *P* = 0.047 in LUAD while r = −0.270, *P* < 0.001 in LUSC; for miR-92a-2, *r* = −0.223, *P* < 0.001 in LUAD while *r* = −0.327, *P* < 0.001 in LUSC; as shown in Figures 5C–F, respectively).

### Expression Level of RGS3 and Its Prognostic Value in NSCLC

**Figure 6:**
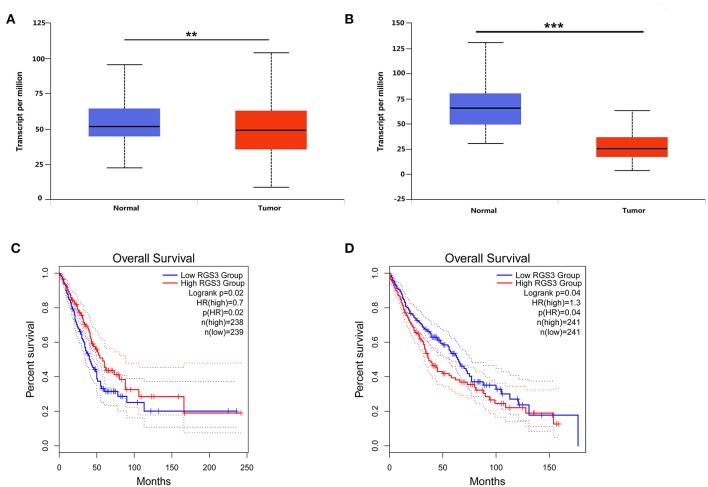
Expression level of RGS3 and its prognostic role in NSCLC. **(A,B)** Expression of RGS3 was downregulated in lung adenocarcinoma and lung squamous cell carcinoma, values are expressed as Transcript per million, **p < 0.01, ***p < 0.001, *t*-test; **(C,D)** The association between expression of RGS3 and the risk of lung adenocarcinoma **(C)** and lung squamous cell carcinoma **(D)**. HR, hazard radio.

To detect the expression level of RGS3 in NSCLC, we downloaded the mRNA expression dataset of RGS3 in NSCLC from TCGA. Results indicated that the expression of miR-92a was low in NSCLC compared with normal tissues (*P* = 0.005 for LUAD, Figure 6A; *P* < 0.001 for LUSC, Figure 6B). Figure 6C described the prognostic role of RGS3 for LUAD with an overall HR of 0.7 (*P* = 0.02), indicating that low-RGS3-expression groups had a higher risk of death probability than the high-expression groups. However, the opposite result was obtained for LUSC as shown in Figure 6D, which demonstrated that high expression level of RGS3 might be a risk factor for the overall survival of LUSC patients.

## Discussion

The identification of non-invasive biomarkers is critical for the early diagnosis, suitable treatment and accurate prognosis of human cancers. MiR-92a family members are involved in multiple biological processes, which are considered to participate in tumorigenesis and tumor progression. Numerous studies have reported ectopic expression of miR-25, miR-92a, and miR-363 have been detected in various tumors, and that they might promote tumor cell proliferation, invasion, and migration and inhibit tumor cell apoptosis. Moreover, a few studies have reported the significant clinical contribution of miR-92a family in cancer diagnosis and prognosis prediction. However, these results were inconsistent and even contradictory due to clinical complexity. The systematic and stratified analyses are necessary to assess the clinical performance of miR-92a family in human cancers.

The present pooling analysis evaluated the clinical value of the miR-92a family for the diagnosis and prognosis of human tumors and the results indicated that the diagnostic accuracy for the entire miR-92a family members was notably high, with the corrected pooled SEN and SPE of 0.85 [0.81–0.88] and 0.86 [0.83–0.90], respectively, while it was 0.92 [0.90–0.94] for AUC. Each single miRNA of miR-92a family also yielded high diagnostic effectiveness with AUC of 0.90 [0.87–0.93] for miR-25, 0.94 [0.92–0.96] for miR-92a and 0.89 [0.82–0.95] for miR-363. In the subgroup analysis, multiple miRNAs had a higher diagnostic value compared to any single miRNA, which indicated that the combination of different miRNAs had higher diagnostic accuracy. The serum miR-25 showed a higher diagnostic value when compared to miR-25 from another specimen, but miR-92a from tissues showed the highest diagnostic effectiveness among all specimens. Interestingly, diagnostic accuracy of miR-25 was higher for the Asian population than the Caucasian population, and it could better distinguish cancer patients from healthy individuals than patients with benign diseases. However, miR-92a had similar cancer detection ability irrespective of ethnicity and types of control. Also, miR-25 could detect lung cancer with AUC of 0.95 [0.93–0.97] and miR-92a could detect gastric cancer with AUC of 0.96 [0.94–0.98]. In colorectal cancer, miR-92a showed a high diagnostic effectiveness with SEN and SPE of 0.85 [95% CI: 0.77–0.91] and 0.89 [95% CI: 0.81–0.94], respectively, and a value of 0.94 [95% CI: 0.91–0.95] for AUC. These results indicated when combined with other miRs, serum miR-25 might be used as an effective early diagnostic biomarker to distinguish patients with lung cancer from healthy individuals in the Asian population, while miR-92a from tissues had potential diagnostic value for patients with gastric cancer, no matter the patient's ethnicity. With respect to the clinical contribution of the miR-92a family to prognosis of cancer patients, the pooled HR for the high and low expression groups was 2.26 [95% CI: 1.70–3.00] indicating that groups with high expression of miR-92a family had an increased risk (2.26 times) of poor outcomes compared to groups with low expression. The group with an upregulated expression of miR-25 and showed an increased risk with respect to disease progression or mortality with the corrected HR of 2.22 [95% CI: 1.67–2.97]. Nevertheless, the overall HR was 2.11 [95% CI: 0.96–4.65] with a Z value of 1.85 and a *P-*value of 0.064 for high expression groups of miR-92a vs. low expression groups, which suggested that these two contrast groups had the same survival probability. All the above conclusions can guide clinical doctors to take the most appropriate diagnosis and treatment measures for different conditions and patients as early as possible.

Since miRNA family members have the same seed area, all family members may have the same target gene. Therefore, we chose the miR-92a as the representative of this family to conduct the follow-up mechanism research. As the result of our research, the expression level of miR-92a was downregulated in LUSC in the TCGA dataset, which was confirmed in NSCLC tissue analysis. We further identified RGS3 as a target gene of miR-92a in lung cancer. The luciferase assay demonstrated that miR-92a could directly bind to the 3′-UTR to decrease the expression of RGS3, one of the “cancer signature” genes owing to its vital role in tumor development. RGS is a GTPase-activating protein, which could directly interact with the alpha subunit of G protein, and then negatively regulate G protein signal conduction by catalyzing the hydrolysis of GTP (Scheschonka et al., 2000). RGS3 belongs to the R4 subfamily of RGS proteins with several homologous isomers including RGS3S, RGS3L, and PDZ-RGS3. RGS3S contains only the RGS domain, which is mainly expressed in the nucleus and can induce apoptosis when overexpressed (Dulin et al., 2000). The other two long isomers, RGS3L and PDZ-RGS3, could interact with Ephrin-B2 receptors to affect cell migration and participate in neuronal formation and axonal orientation (Qiu et al., 2010). Meanwhile, PDZ-RGS3 can enhance the typical Wnt signaling pathway and promote the progress of epithelial mesenchymal transformation (EMT) (Shi et al., 2012). The overexpressed RGS3 in the tissues of patients with gastric cancer could lead to a poor prognosis, which could be negatively regulated by miR-126 (Wang et al., 2017). In addition, the abnormal expression of RGS3 might regulate the TGF-β signaling pathway by interfering with the heteromerization of Smad protein (Xu et al., 2017). Lu S et al. reported that the overexpression of HOXD-AS1 in human hepatocyte tumors negatively regulated the expression level of RGS3, thereby inhibiting Dox-induced apoptosis (Lu S. et al., 2017), while overexpression of RGS3 in glioma cells promotes cell adhesion and metastasis (Tatenhorst et al., 2004).

As far as we know, this research is the first one to summarize the clinical value of the miR-92a family on cancer diagnosis and prognosis though there have been several meta-analyses data published that focused on the correlation between a single miRNA and its clinical application. In one study dedicated to the role of miR-92a in colorectal cancer diagnosis, the authors drew a conclusion from only six publications, suggesting miR-92a might be a biomarker for colorectal cancer with moderate detection ability, with SEN and SPE of 0.76 [0.72–0.79] and 0.64 [0.59–0.69], respectively (Yang et al., 2014). Based on a large number of researches and participants in this work, we thought miR-92a could serve as a non-invasive and convenient biomarker for the detection of colorectal cancer with high sensitivity and specificity of 0.85 [95% CI: 0.77–0.91] and 0.89 [95% CI: 0.81–0.94], respectively. Another research conducted by Qu et al., showed higher expression of miR-25 could predict a worse outcome with the pooled HR of 2.434 [95% CI: 1.330–3.539, *P* < 0.001] when compared to lower expression groups (Qu et al., 2015). This result was consistent with our results which supported a positive association between miR-25 expression and the risk of poor outcome, in human cancers.

Despite the above efforts and advantages of this study, some limitations in the present study still need to be addressed. The first and most important one was heterogeneity among the included articles, which could not be neglected for any meta-analysis, and which might potentially influence the final results. Therefore, we employed the meta-regression and subgroup analysis with a random effect model to reduce or avoid the influence of heterogeneity. Secondly, due to insufficient data on cancer types and studies, subgroup analyses of prognostic meta-analyses could not be conducted. Moreover, some studies might have been omitted in the literature selection process. All these above considerations may lead to negative conclusions; therefore, further larger, long-term follow-up studies are needed to obtain robust and possibly definitive evidence.

In summary, this study demonstrated that serum miR-25 might play an important role in a clinical setting for cancer early diagnosis and prognosis prediction, especially for lung cancer in an Asian population; while tissue miR-92a may be a suitable biomarker for cancer detection, and this family might function through targeting RGS3.

## Data Availability Statement

All datasets generated for this study are included in the manuscript/Supplementary Files.

## Ethics Statement

The studies involving human participants were reviewed and approved by Institutional Review Board of China Medical University. The patients/participants provided their written informed consent to participate in this study.

## Author Contributions

BZ and MJ planned the research. MJ and XiL retrieved literatures. MJ finished the experiments. XuL and XQ conducted statistical analysis. BZ, XuL, and MJ wrote and edited this article.

### Conflict of Interest

The authors declare that the research was conducted in the absence of any commercial or financial relationships that could be construed as a potential conflict of interest.

## Abbreviations

95% CI: 95% confidence interval
AUC: the area under the SROC curve
BD: benign disease
C13orf25: chromosome 13 open reading frame 25
DFS: disease free survival
DOR: diagnostic odds ratio
EMT: epithelial mesenchymal transformation
FBS: fetal bovine serum
FN: false negative
FP: false positive
HC: healthy control
HR: hazard ratio
LUAD: lung adenocarcinoma
LUSC: lung squamous cell carcinomas
mcm7: minichromosome maintenance protein 7
MFS: metastasis free survival
NLR: negative likelihood ratio
NOS: the Newcastle-Ottawa Scale
OS: overall survival
NSCLC: Non-small-cell lung cancer
PFS: progression-free survival
PRISMA: Preferred Reporting Items for Systematic Reviews and Meta-Analyses
PLR: positive likelihood ratio
qRT-PCR: quantitative real-time polymerase chain reaction
QUADAS-2: the Quality Assessment of Diagnostic Accuracy Studies-2
RFS: relapse free survival
RGS3: the regulator of G-protein signaling 3
RPMI: Roswell Park Memorial Institute
SEN: sensitivity
SPE: specificity
SROC: summary receiver operating characteristic
TN: true negative
TP: true positive.
