# Supplementary material for: MiR-92a Family: A Novel Diagnostic Biomarker and Potential Therapeutic Target in Human Cancers
Source: Front Mol Biosci. 2019 Oct 1;6:98. doi: 10.3389/fmolb.2019.00098 (PMC6779690; doi:10.3389/fmolb.2019.00098)
Supplement: Supplementary file 1 [file Data_Sheet_1.pdf]

MiR-92a family: A Novel Diagnostic Biomarker and Potential Therapeutic Target in Human Cancers

Min Jiang[1], Xuelian Li[1], Xiaowei Quan[1], Xiaoying Li[1], Baosen Zhou[1]*

1 Department of Epidemiology, School of Public Health, China Medical University, Shenyang, People’s Republic of China.

* Correspondence:

Baosen Zhou

No. 77 Puhe Road, Shenyang North New Area, Shenyang, Liaoning province, 110122, People’s Republic of China.

Tel./Fax: +86-133-8688-1563

bszhou@cmu.edu.cn

Supplementary appendix to the manuscript

Table S1 main characteristics of enrolled studies in diagnostic meta-analyses

Study ID	ethnicity	specimen	case		control		Cancer-type	Control-type	stage	miRNA	Reference miRNA	SEN (%)	SPE (%)
			N	age	N	age							
**MiR-92a**													
Liu HN 2018	Asian	serum	85	59.5	78	34.8	CRC	HC	Ⅰ-Ⅳ	miR-92a, miR-29a, miR-21, miR-125, miR-223	cel-miR-39	84.7	98.7

Liu HN 2018	Asian	serum	85	59.5	78	34.8	CRC	HC	Ⅰ-Ⅳ	miR-92a	cel-miR-39	78.8	71.8
Elshafei A 2017	Oceanian	serum	64	51.4	27	46.4	CRC	HC	Ⅰ-Ⅳ	miR-92a	SNORD-68	84.4	100.0
Elshafei A 2017	Oceanian	serum	32	51.8	27	46.4	CRC	HC	I-Ⅱ	miR-92a	SNORD-68	78.1	100.0
Elhamamsy A 2017	African	plasma	65	55.3	50	54.7	AML	HC	NA	miR-92a	U6	81.5	94.0
MotawKi TK 2016	Oceanian	plasma	70	56.2	62	57.2	BLC	HC	Ⅱ-Ⅲ	miR-92a	U6	97.0	76.7
MotawKi TK 2016	Oceanian	plasma	70	56.2	62	57.2	BLC	HC	Ⅱ-Ⅲ	miR-92a,miR-100 and miR-143	U6	94.3	83.3
Chang PY 2016	Asian	plasma	353	62.9	492	52.5	CRC	HC	Ⅰ-Ⅳ	miR-92a,miR-223	cel-miR-238	96.8	75.0
Fan LH 2016	Asian	serum	94	60.5	58	58.1	NSCLC	HC	Ⅰ-III	miR-92a	-	86.8	65.5
Huang S 2016	Asian	serum	30	NA	30	NA	GC	HC	Ⅰ-Ⅳ	miR-21,miR-31, miR-92a, miR-181b, miR-203	miR-16	90.0	96.7
Huang S 2016	Asian	serum	62	NA	59	NA	GC	HC	Ⅰ-Ⅳ	miR-21,miR-31, miR-92a, miR-181b, miR-203	miR-16	85.5	98.3
Wen Y 2015	Asian	plasma	67	48.5	82	48.6	HCC	BD	NA	miRNAs[a]	cel-miR-39	86.6	64.6
Zhu C 2014	Asian	plasma	40	53.8	40	53.6	GC	HC	I-Ⅱ	miR-92a	cel-miR-39	97.5	85.0
Zhu C 2014	Asian	plasma	40	53.8	40	53.6	GC	HC	I-Ⅱ	miR-16, miR-25, miR-92a, miR-451, miR-486-5p	cel-miR-39	97.5	87.5
Zhu C 2014	Asian	plasma	48	56.6	102	54.0	GC	HC,BD	I-Ⅱ	miR-92a	cel-miR-39	72.9	73.5
Zhu C 2014	Asian	plasma	48	56.6	102	54.0	GC	HC,BD	I-Ⅱ	miR-16, miR-25, miR-92a, miR-451, miR-486-5p	cel-miR-39	72.9	89.2
Du ML 2014	Asian	plasma	49	61.1	49	61.7	CRC	HC	Ⅱ-Ⅲ	miR-92a	cel-miR-39	18.4	95.9
Zheng G 2014	Asian	serum	117	56.3	175	52.6	CRC	BD,HC	Ⅰ-Ⅳ	miR-19a-3p, miR-223-3p, miR-92a, miR-422a	miR-191-5p, U6	84.3	91.6

Wang J 2014	Asian	serum	30	NA	30	NA	CRC	HC	Ⅰ-Ⅳ	miR-21, let-7g, miR-31, miR-92a, miR-181b, miR-203	miR-16	83.3	96.7
Wang J 2014	Asian	serum	83	NA	59	NA	CRC	HC	Ⅰ-Ⅳ	miR-21, let-7g, miR-31, miR-92a, miR-181b, miR-203	miR-16	96.4	88.1
Liu GH 2013	Asian	serum	200	NA	80	NA	CRC	HC	Ⅰ-Ⅳ	miR-92a, miR-21	miR-16	68.0	91.2
Liu GH 2013	Asian	serum	200	NA	80	NA	CRC	HC	Ⅰ-Ⅳ	miR-92a	miR-16	65.5	82.5
Wang SY 2013	Asian	tissue	25	NA	21	NA	CRC	BD	NA	miR-375, miR-424, miR-92a	U47	92.0	95.0
Wang SY 2013	Asian	tissue	25	NA	20	NA	CRC	BD	NA	miR-375, miR-424, miR-92a	U47	96.0	90.0
Wang SY 2013	Asian	tissue	33	NA	25	NA	CRC	BD	NA	miR-375, miR-424, miR-92a	U47	94.0	84.0
Luo XY 2013	Caucasian	plasma	80	68.0	144	62.5	CRC	BD	Ⅰ-Ⅳ	miR-92a	miR-16	68.2	49.4
Torres A 2013	Caucasian	tissue	73	62.8	31	44.8	EEC	BD	Ⅰ-Ⅳ	miR-92a	U48, U44, U75	78.0	90.0
Torres A 2013	Caucasian	tissue	73	62.8	31	44.8	EEC	BD	Ⅰ-Ⅳ	miR-92a, miR-205,miR-410	U48, U44, U75	96.0	100.0
Torres A 2013	Caucasian	plasma	34	60.2	14	54.7	EEC	HC	Ⅰ-Ⅳ	miR-92a	miRNAs[b]	61.0	93.0
Torres A 2013	Caucasian	plasma	34	60.2	14	54.7	EEC	HC	Ⅰ-Ⅳ	miR-92a,miR-9	miRNAs[b]	73.0	100.0
Wu CW 2012	Asian	stool	88	67.2	101	60.5	CRC	HC	Ⅰ-Ⅳ	miR-92a, miR-21	U6	81.8	57.4
Wu CW 2012	Asian	stool	88	67.2	101	60.5	CRC	HC	Ⅰ-Ⅳ	miR-92a	U6	71.6	73.3
Wang QF 2012	Asian	plasma	90	62.0	58	58.0	CRC	HC	Ⅰ-Ⅳ	miR-92a, miR-29a, miR-760	cel-miR-39	83.3	93.1
Baraniskin A 2011	Caucasian	CF	23	64.0	30	NA	PCNSL	BD	NA	miR-92a	miR-24	95.7	80.0
Ng EK 2009	Asian	plasma	90	NA	50	NA	CRC	HC	Ⅰ-Ⅳ	miR-92a	U6	89.0	70.0
Huang ZH 2009	Asian	plasma	100	61.0	59	58.0	CRC	HC	Ⅰ-Ⅳ	miR-92a, miR-29a	miR-16	83.0	84.7

Huang ZH 2009	Asian	plasma	100	61.0	59	58.0	CRC	HC	Ⅰ-Ⅳ	miR-92a	miR-16	84.0	71.2
**MiR-25**													
Li FX 2017	Asian	plasma	65	54.1	65	56.2	GC	HC	Ⅰ-Ⅳ	miR-25	U6	87.6	76.9
Le Q 2017	Asian	serum	41	61.0	41	58.0	GC	HC	NA	miR-25	U6	78.1	61.0
Fujiwara1 T 2017	Asian	serum	14	NA	22	NA	OS	HC,BD	NA	miR-25	cel-miR-39	71.4	92.3
JohansenJS 2016	Caucasian	serum	133	NA	51	NA	PC	HC	Ⅰ-Ⅳ	miR-16,miR-27a,miR-25,miR-29c, miR-483-5p	miR-292, miR-259	85.0	33.0
JohansenJS 2016	Caucasian	serum	198	NA	153	NA	PC	HC	Ⅰ-Ⅳ	miR-16,miR-27a,miR-25,miR-29c, miR-483-5p	miR-292, miR-259	85.0	66.0
JohansenJS 2016	Caucasian	serum	86	NA	44	NA	PC	HC	Ⅰ-Ⅳ	miR-16,miR-27a,miR-25,miR-29c, miR-483-5p	miR-292, miR-259	85.0	67.0
Deng T 2016	Asian	serum	303	62.0	600	49.0	PC	HC	Ⅰ-Ⅳ	miR-25	U6, 5S rRNAs	75.6	93.0
Cochetti G 2016	Caucasian	serum	64	63.0	60	NA	PRC	BD	NA	miR-25	miR-191-5p, miR-425-5p	38.5	88.0
Cappellesso R 2016	Caucasian	cytologic	29	73.2	24	61.2	MM	BD	NA	miR-25	U6	54.0	72.0
Bryzgunova O 2016	Caucasian	Urine exosome	14	72.0	20	59.0	PRC	HC	NA	miR-25	miR-16	71.0	60.0
Butz H 2016	Caucasian	urine exsome	28	59.0	18	NA	RCC	HC	NA	miR-126-3p,miR-25	miR-16,miR-106a	62.1	85.0
Butz H 2016	Caucasian	urine exsome	81	NA	33	NA	RCC	HC	NA	miR-126-3p,miR-25	miR-16,miR-106a	52.5	86.2
Zhou X 2015	Asian	plasma	101	NA	91	NA	GC	HC	Ⅰ-Ⅳ	miR-185, miR-20a, miR-210,	cel-miR-39,	65.0	80.0

										miR-25, miR-92b	U6		
Wen Y 2015	Asian	plasma	67	48.5	82	48.6	HCC	BD	NA	miR-25	cel-miR-39	55.3	79.3
Wen Y 2015	Asian	plasma	67	48.5	82	48.6	HCC	BD	NA	miR-20a-5p, miR-25,miR-30a-5p, miR-92a-3p, miR-132-3p, miR-185-5p, miR-320a, miR-324-3p	cel-miR-39	86.6	64.6
Wang C 2015	Asian	serum	19	62.1	19	61.8	NSCLC	HC	Ⅰ-Ⅳ	miR-483-5p,miR-193a-3p, miR-25, miR-214, miR-7	let-7d/g/i	100.0	84.0
Wang C 2015	Asian	serum	63	59.7	63	61.9	NSCLC	HC	Ⅰ-Ⅳ	miR-483-5p,miR-193a-3p, miR-25, miR-214, miR-7	let-7d/g/i	89.0	68.0
Wang C 2015	Caucasian	serum	108	67.2	48	58.5	NSCLC	HC	Ⅰ-Ⅳ	miR-483-5p,miR-193a-3p, miR-25, miR-214, miR-7	let-7d/g/i	95.0	84.0
Wang C 2015	Caucasian	serum	108	67.2	56	63.7	NSCLC	BD	Ⅰ-Ⅳ	miR-483-5p,miR-193a-3p, miR-25, miR-214, miR-7	let-7d/g/i	95.0	95.0
Meng XD 2015	Caucasian	serum	180	60.0	66	59.0	EOC	HC	Ⅰ-Ⅳ	miR-7, miR-25, miR-93, miR-429	cel-miR-39	93.0	92.0
Meng XD 2015	Caucasian	serum	180	60.0	66	59.0	EOC	HC	Ⅰ-Ⅳ	miR-25	cel-miR-39	92.4	63.9
Li M 2015	Asian	plasma	56	NA	95	NA	PTC	BD	Ⅰ-Ⅳ	miR-25	U6	92.8	68.8
Jia WH 2015	Asian	serum	123	46.0	94	47.8	CC	HC	Ⅰ-Ⅳ	miR-21, miR-29a, miR-25, miR-200a, miR-486-5p	let-7d/j/i	81.0	88.6
Wang P 2015	Asian	serum	142	61.1	111	59.8	LC	HC	Ⅰ-Ⅳ	miR-125a-5p, miR-25, miR-126	cel-miR-39	88.0	82.6
Wang P 2015	Asian	serum	94	NA	111	59.8	LC	HC	I-Ⅱ	miR-125a-5p, miR-25, miR-126	cel-miR-39	87.5	87.5
Wang P 2015	Asian	serum	94	NA	111	59.8	LC	HC	I-Ⅱ	miR-25	cel-miR-39	83.3	75.0

Zhu C 2014	Asian	plasma	40	53.8	40	53.6	GC	HC	I-Ⅱ	miR-25	cel-miR-39	87.5	97.5
Zhu C 2014	Asian	plasma	40	53.8	40	53.6	GC	HC	I-Ⅱ	miR-16, miR-25, miR-92a, miR-451, miR-486-5p	cel-miR-39	97.5	87.5
Zhu C 2014	Asian	plasma	48	56.6	102	54.0	GC	HC,BD	I-Ⅱ	miR-25	cel-miR-39	41.7	97.1
Zhu C 2014	Asian	plasma	48	56.6	102	54.0	GC	HC,BD	I-Ⅱ	miR-16, miR-25, miR-92a, miR-451, miR-486-5p	cel-miR-39	72.9	89.2
Wu CY 2014	Asian	serum	63	62.0	63	59.7	ESCC	HC	Ⅰ-Ⅳ	miR-25	let-7d/g/i	81.0	81.0
Pan WZ 2014	Asian	plasma	30	58.4	26	NA	PC	HC	Ⅰ-Ⅳ	miR-25	cel-miR-39	73.3	73.1
Mengual LV 2013	Caucasian	urine	151	72.0	126	63.0	BLC	HC,BD	NA	miR-25, miR-18a, miR-187, miR-204,miR-142-3p, miR-140-3p	miR-103, miR-30c	84.8	86.5
Hu ZB 2012	Asian	serum	48	54.0	48	51.0	BC	HC	Ⅰ-Ⅲ	miR-16, miR-25, miR-222, miR-324-3p	miR-484, miR-191	91.7	89.6
Hu ZB 2012	Asian	serum	76	53.0	76	49.2	BC	HC	Ⅰ-Ⅲ	miR-16, miR-25, miR-222, miR-324-3p	miR-484, miR-191	92.1	93.4
li LM 2010	Asian	serum	55	52.8	50	47.7	HCC	HC	NA	miR-375, miR-25,let-7f	-	97.9	99.1
**MiR-363**													
Wu CW 2017	Caucasian	blood	29	61.2	115	66.2	CRC	HC	Ⅰ-Ⅳ	miR-144-3p, miR-451a, miR-144-5p, miR-20b-5p, miR-486-5p, miR-363	cel-miR-39	66.0	95.0

a: miR-20a-5p, miR-25-3p, miR-30a-5p, miR-92a-3p, miR-132-3p, miR-185-5p, miR-320a, miR-324-3p.

b: miR-93, miR-26b, miR-192, miR-103a, miR-142-3p.

Abbreviation: AML, acute myeloid leukemia; BC, breast cancer; BLC, bladder cancer; CC, cervical cancer; CF, cerebrospinal fluid; CRC, colorectal cancer; EEC, endometrioid endometrial carcinoma; EOC, epithelial ovarian cancer; ESCC, esophageal squamous cell carcinoma; GC, gastric cancer; HCC, hepatocellular carcinoma; LC, lung cancer; LUAD, lung adenocarcinomas; MM, malignant mesothelioma; N, number; NMIBC, non-muscle invasive bladder cancer; NSCLC, non-small cell lung cancer; OS, osteosarcoma; PCNSL, primary central nervous system lymphoma; PRC, prostate cancer; PTC, papillary thyroid carcinoma; RCC, renal cell carcinoma; SEN, sensitivity; SPE, specificity.

Table S2 main characteristics of enrolled researches in prognostic meta-analyses

**Study ID**	**No. of low**	**No. of high**	**ethnicity**	**specimen**	**cancer**	**stage**	**miRNA**	**reference miRNA**	**outcome**	**Median Follow-up (month)**	**HR**	**LL**	**UL**	NOS
**MiR-92a**														
Ingelmo-Torres M 2017	NA	NA	Caucasian	Urinary cell	NMIBC	NA	miR-92a	miR-103-3p, miR-30c-5p	PFS	47	6.210	2.090	18.450	7
Ren CL 2016	34	146	Asian	tissue	GC	Ⅰ-Ⅳ	miR-92a	Scramble-miR	OS	85.2	3.340	1.670	6.700	6

Yang W 2015	53	53	Asian	tissue	HCC	Ⅰ - Ⅳ	miR-92a	U6	OS	38,7	2.283	1.104	4.717	8
Zhou T 2013	41	41	Asian	tissue	CRC	Ⅰ - Ⅳ	miR-92a	U6	OS	NA	2.342	1.072	5.115	7
Nilsson S 2012	59	58	Caucasian	tissue	BC	Ⅰ -III	miR-92a	NA	RFS	78	0.375	0.145	0.972	7
**MiR-25**														
Jacob H 2017	48	63	Caucasian	tissue	CRC	II-III	miRNAs[a]	miRNAs[b]	DFS	NA	21.40	4.210	108.700	7
Jacob H 2017	90	119	Caucasian	tissue	CRC	II-III	miRNAs[a]	miRNAs[b]	DFS	NA	2.000	1.040	3.890	8
Goossens-Beumer I 2015	29	21	Caucasian	tissue	CRC	II-III	miR-25	miR-16-5p,miR-26a-5p	RFS	51.2	2.370	0.930	6.030	8
Goossens-Beumer I 2015	26	17	Caucasian	tissue	CRC	II-III	miR-25	miR-16-5p,miR-26a-5p	RFS	83.5	5.760	1.820	18.200	8
Wu CY 2014	12	51	Asian	serum	ESCC	Ⅰ - Ⅳ	miR-25	let-7d/g/i	OS	19.6	3.840	1.020	14.410	6
Wu CH 2014	73	121	Asian	serum	ESCC	Ⅰ - Ⅳ	miR-25	U6, miR-16	OS	31.3	1.127	0.778	1.637	7
Wang X 2014	NA	NA	Asian	tissue	EOC	Ⅰ - Ⅳ	miR-25	U6	OS	NA	2.119	1.568	3.221	6
Su ZX 2014	NA	NA	Asian	tissue	HCC	Ⅰ - Ⅳ	miR-25	U6	OS	47	2.179	1.876	4.335	6
Li XJ 2014	78	108	Asian	tissue	CRC	Ⅰ -	miR-25	U44	OS	NA	1.900	1.220	2.970	8

						Ⅳ								
Xu FX 2014	49	51	Asian	tissue	LUAD	Ⅰ - Ⅳ	miR-25	U6	OS	34	2.340	1.020	5.350	6
Jonsdottir K 2012	119	84	Caucasian	tissue	BC	NA	miR-25	U6	MFS	122	2.400	1.100	5.300	8

a: miR-143-5p, miR-27a-3p, miR-31-5p, miR-181a-5p, miR-30b-5p, miR-30d-5p, miR-146a-5p,miR-23a-3p, miR-150-5p, miR-210-3p, miR-25-3p, miR-196a-5p, miR-148a-3p, miR-222-3p, miR-30c-5p and miR-223-3p; b: UniSp3,cel-miR-39-3p, UniSp2, UniSp4, UniSp5, UniSp6; Abbreviation: HR, hazard ratio; LL, lower limit; UL, upper Limit; NOS, Newcastle-Ottawa Scale; NMIBC, non-muscle invasive bladder cancer; GC, gastric cancer; HCC, hepatocellular carcinoma; CRC, colorectal cancer; BC, breast cancer; ESCC, esophageal squamous cell carcinoma; EOC, epithelial ovarian cancer; LUAD, lung adenocarcinoma; OS, overall survival; PFS, progression-free survival; RFS, relapse free survival; DFS, disease free survival; MFS, metastasis free survival.

Table S3 NOS scores for each study included in the prognosis meta-analysis

Items	Representativeness of the exposed cohort（1）	Selection of the non-exposed cohort（1）	Ascertainment of exposure（1）	Demonstration that outcome of interest was not present at start of study（1）	Comparability of cohorts on the basis of the design or analysis（2）	Assessment of outcome（1）	Was follow-up long enough for outcomes to occur（1）	Adequacy of follow up of cohorts（1）	total scores（9）
Jacob H 2017	0	1	1	1	1	1	1	1	7
Jacob H 2017	0	1	1	1	2	1	1	1	8
Ingelmo-Torres M 2017	0	1	1	1	1	1	1	1	7
Ren CL 2016	0	0	1	1	1	1	1	1	6
Yang W 2015	1	1	1	1	1	1	1	1	8
Goossens-Beumer I 2015	1	1	1	1	1	1	1	1	8
Wu CY2014	1	1	1	1	1	1	0	0	6
Wu CH 2014	1	1	1	1	1	1	0	1	7
Wang X 2014	1	0	1	1	1	0	1	1	6
Su ZX 2014	1	0	1	1	1	0	1	1	6
Li XJ 2014	1	1	1	1	1	1	1	1	8
Zhou T 2013	1	0	1	1	1	1	1	1	7
Nilsson S 2012	1	0	1	1	1	1	1	1	7

Jonsdottir K 2012	1	1	1	1	1	1	1	1	8
Xu FX 201	1	1	1	1	1	1	0	0	6
Ranade AR 2010	1	1	1	1	0	1	1	1	7

NOS, newcastle - ottawa quality assessment.

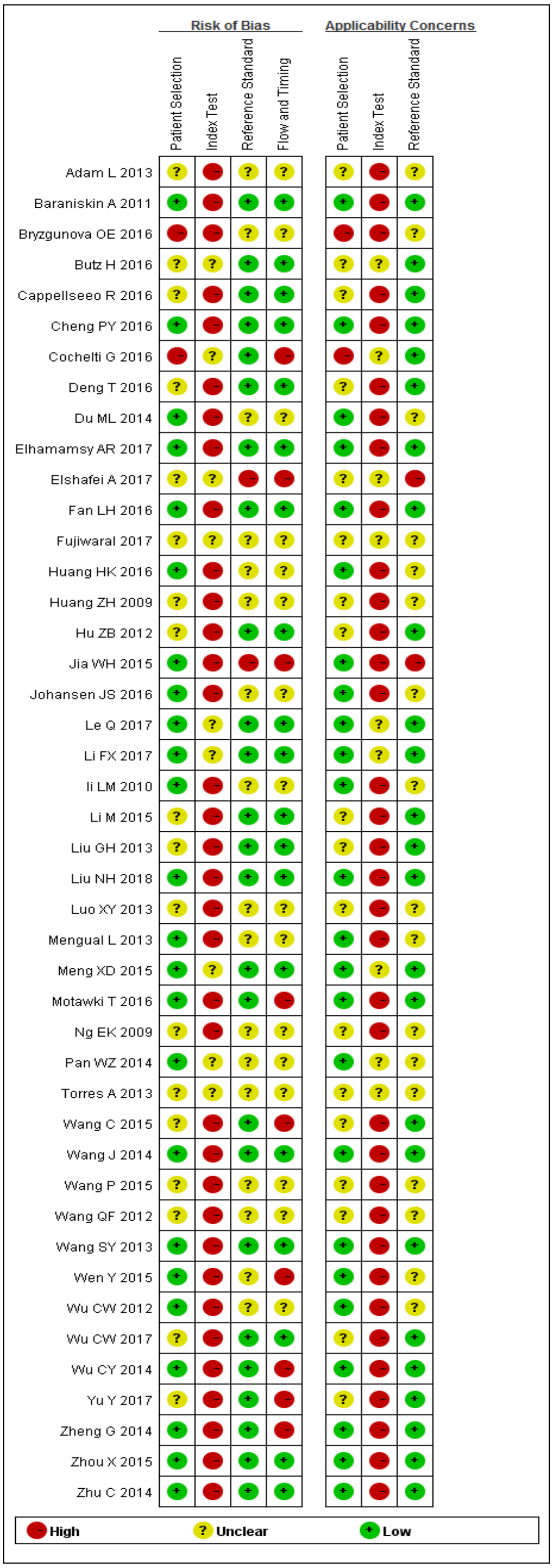

Figure S1: Risk of bias graph: a plot of the distribution of judgments (Yes, No, Unclear) across studies for each

risk of bias entry for each enrolled literature in the diagnostic meta-analysis.

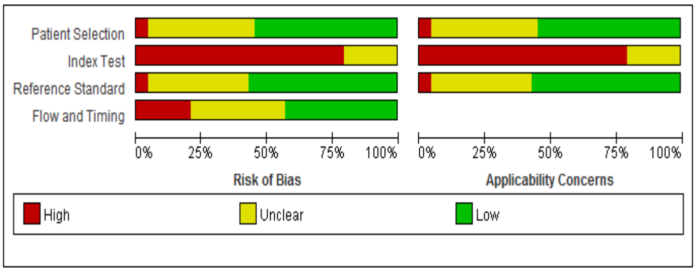

Figure S2: Risk of bias summary: a summary table of review authors' judgments for each risk of bias entry for each study in the diagnostic meta-analysis: proportion of articles with risk of bias (left) and proportion of articles with regarding applicability (right).

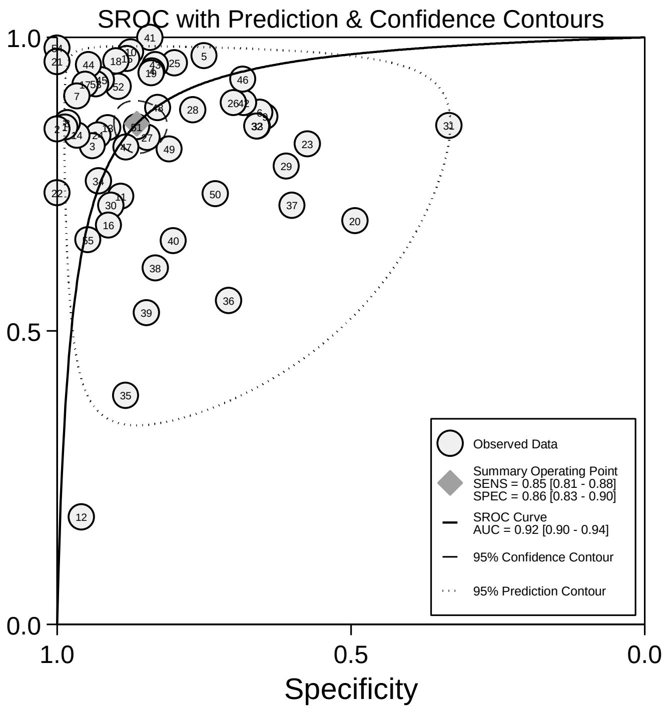

Figure S3: SROC curve of the whole miR-92a family for cancer diagnostic meta-analysis. SENS, sensitivity; SPEC, specificity; SROC, summary receiver operating characteristic; AUC, the area under the SROC curve.

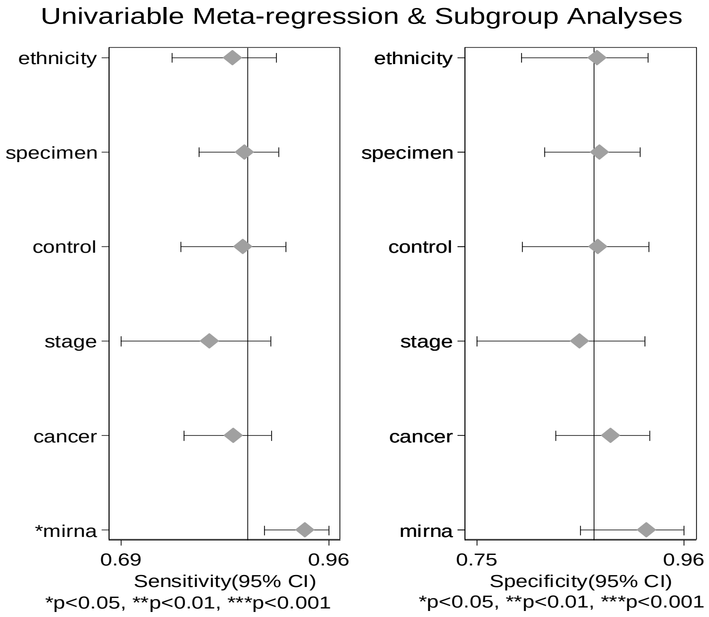

Figure S4: Forest plots for the diagnostic meta-regression analysis: sensitivity (left) and specificity (right). The factors included ethnicity, specimen, control types (healthy controls or patients with benign diseases), stage, cancer types and miRNA-profilings (single miRNA or multiple miRNAs)

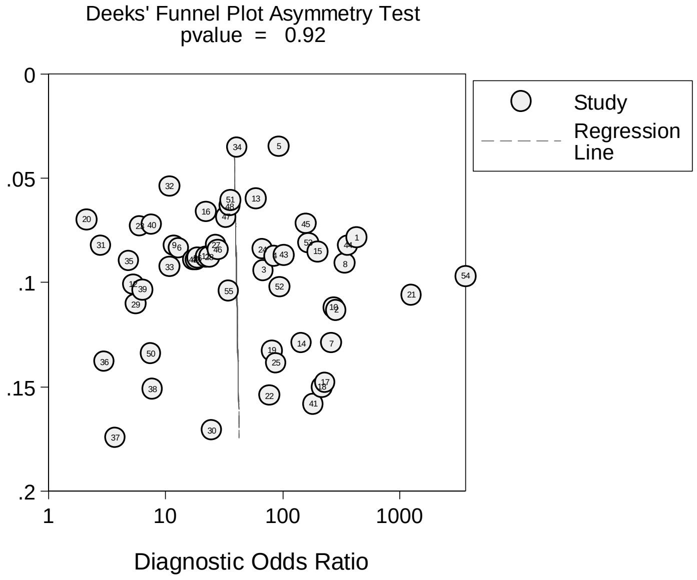

Figure S5: The Deek's test for assessing the publication bias for miR-92a family in the detection of human cancers.

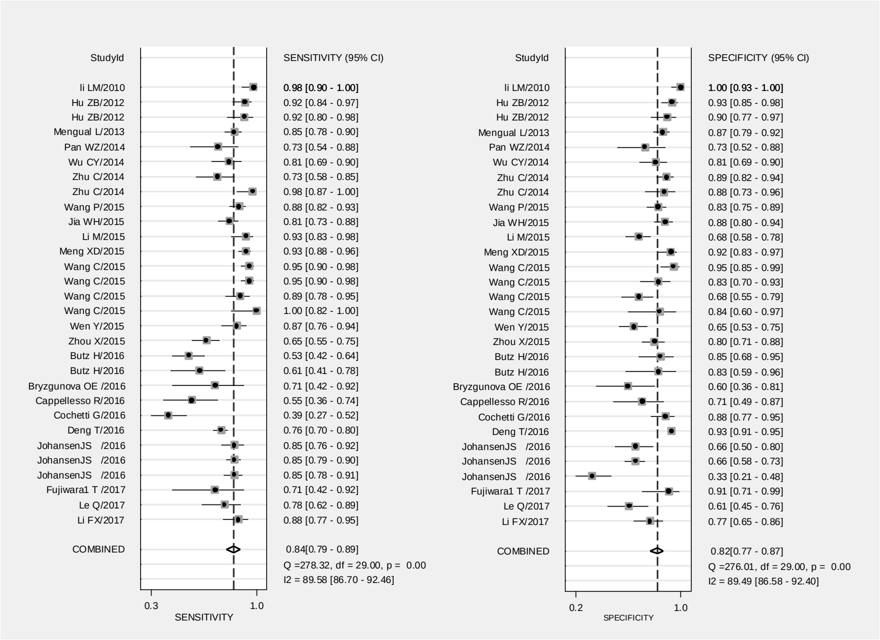

Figure S6: Forest plots of sensitivity and specificity for the cancer diagnosis of miR-25. Both the sensitivity and specificity of each study were showed by square with its 95% Confidence interval showed by the error bars.

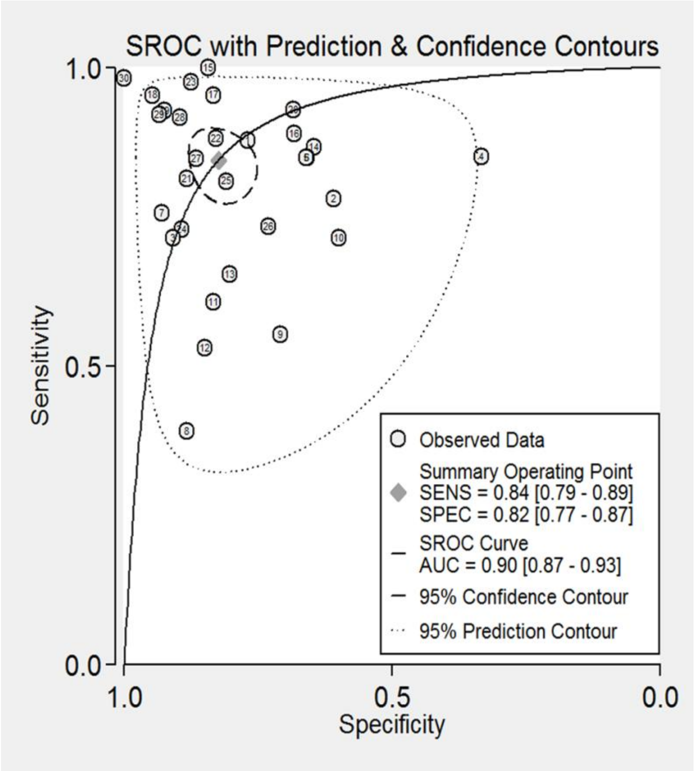

Figure S7: SROC curve of miR-25 for cancer diagnostic meta-analysis. SENS, sensitivity; SPEC, specificity; SROC, summary receiver operating characteristic; AUC, the area under the SROC curve.

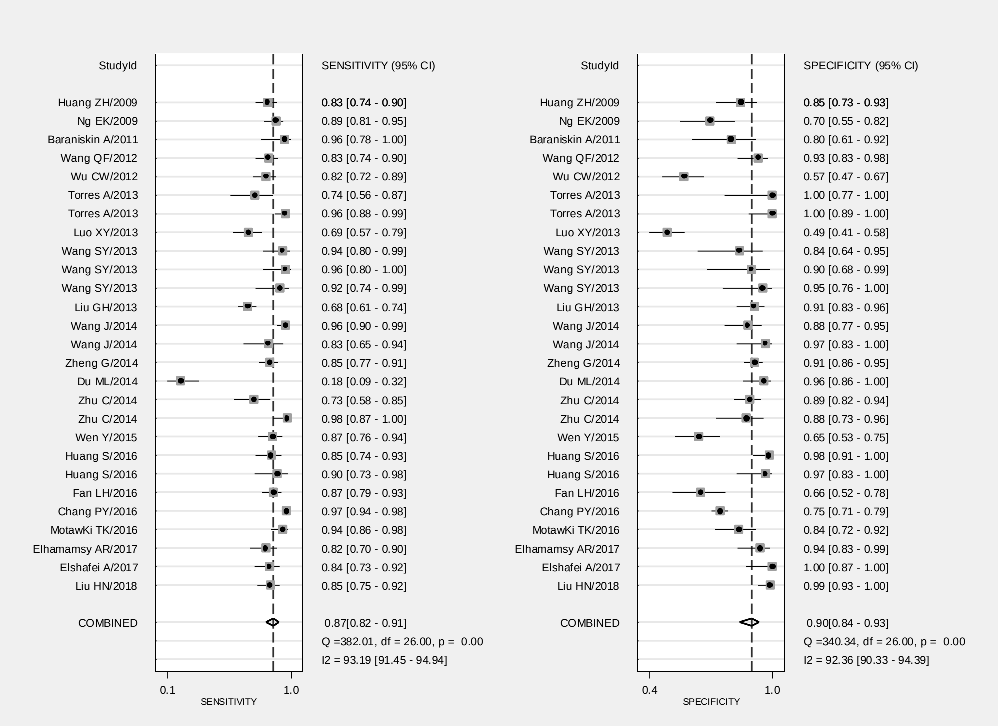

Figure S8: Forest plots of sensitivity and specificity for the cancer diagnosis of miR-92a. Both the sensitivity and specificity of each study were showed by square with its 95% Confidence interval showed by the error bars.

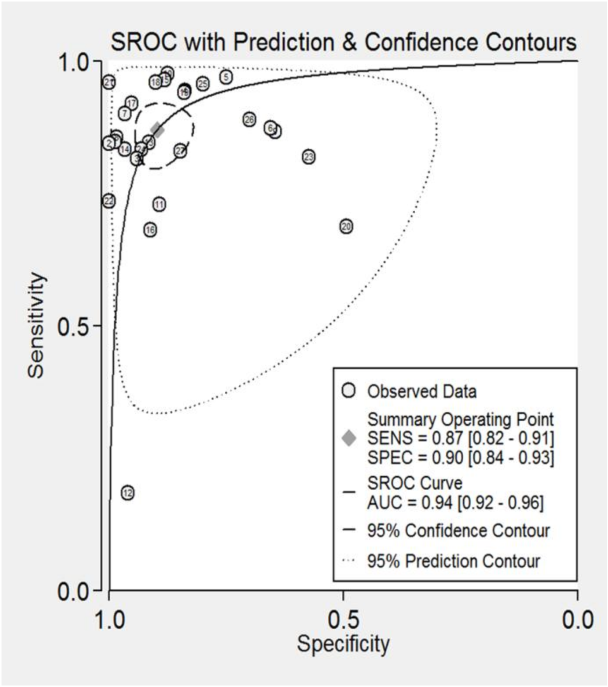

Figure S9: SROC curve of miR-92a for cancer diagnostic meta-analysis. SENS, sensitivity; SPEC, specificity; SROC, summary receiver operating characteristic; AUC, the area under the SROC curve.

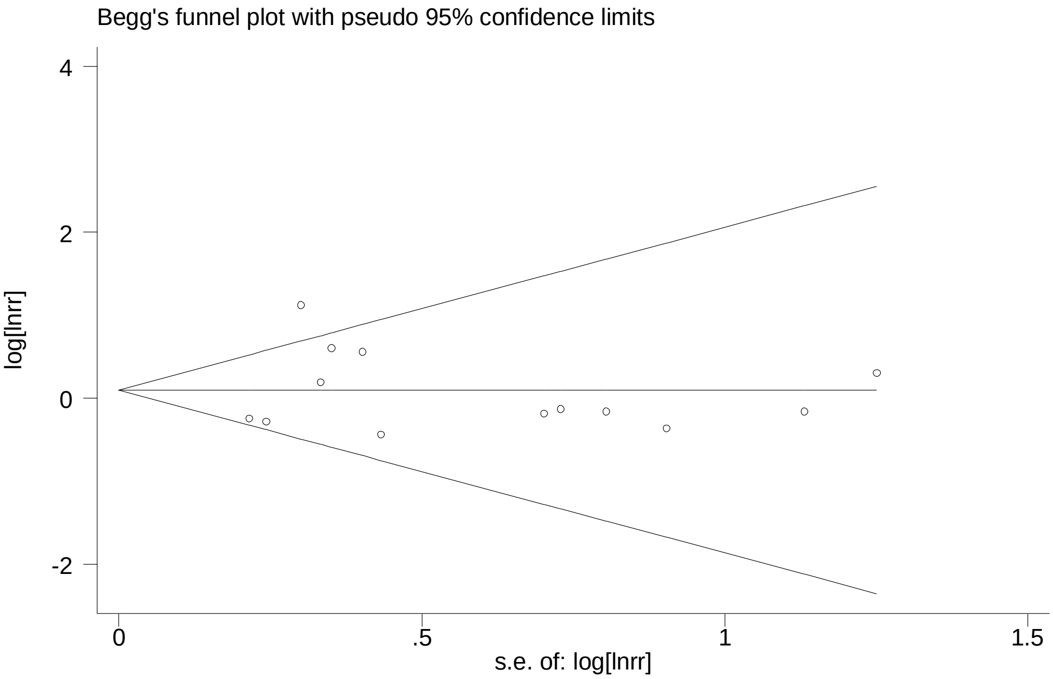

Figure S10: The Begg’s funnel plot to assess the publication bias for miR-92a family in cancer prognosis prediction.

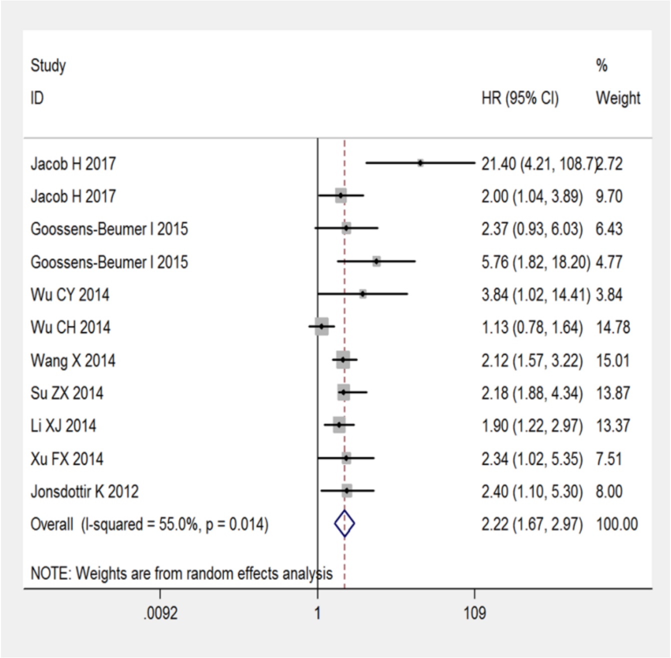

Figure S11: Forest plot of the prognostic meta-analysis of the association between miR-25 and the risk of cancers. HR, hazard radio; 95% CI, 95% confidence interval.

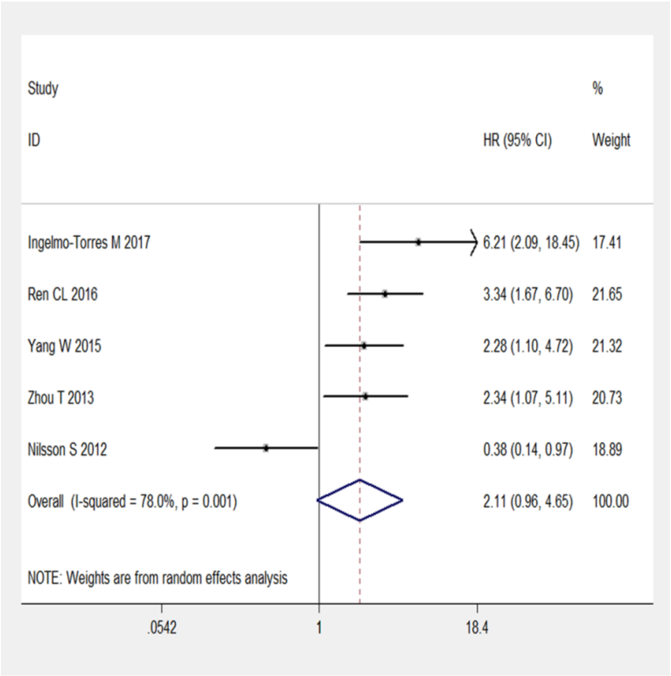

Figure S12: Forest plot of the prognostic meta-analysis of the association between miR-92a and the risk of cancers. HR, hazard radio; 95% CI, 95% confidence interval.

Reference

1. Liu HN, Liu TT, Wu H, et al. Serum microRNA signatures and metabolomics have high diagnostic value in colorectal cancer using two novel methods. *Cancer science.* 2018;109(4):1185-1194.
2. Elshafei A, Shaker O, Abd El-Motaal O, Salman T. The expression profiling of serum miR-92a, miR-375, and miR-760 in colorectal cancer: An Egyptian study. *Tumour biology : the journal of the International Society for Oncodevelopmental Biology and Medicine.* 2017;39(6):1010428317705765.
3. Elhamamsy AR, El Sharkawy MS, Zanaty AF, Mahrous MA, Mohamed AE, Abushaaban EA. Circulating miR-92a, miR-143 and miR-342 in Plasma are Novel Potential Biomarkers for Acute Myeloid Leukemia. *International journal of molecular and cellular medicine.* 2017;6(2):77-86.
4. Motawi TK, Rizk SM, Ibrahim TM, Ibrahim IA. Circulating microRNAs, miR-92a, miR-100 and miR-143, as non-invasive biomarkers for bladder cancer diagnosis. *Cell biochemistry and function.* 2016;34(3):142-148.
5. Chang PY, Chen CC, Chang YS, et al. MicroRNA-223 and microRNA-92a in stool and plasma

samples act as complementary biomarkers to increase colorectal cancer detection. *Oncotarget.* 2016;7(9):10663-10675.

6. Fan L, Qi H, Teng J, et al. Identification of serum miRNAs by nano-quantum dots microarray as diagnostic biomarkers for early detection of non-small cell lung cancer. *Tumor Biology.* 2016;37(6):7777-7784.
7. Huang S, Wang J, Li J, et al. Serum microRNA expression profile as a diagnostic panel for gastric cancer. *Japanese journal of clinical oncology.* 2016;46(9):811-818.
8. Wen Y, Han J, Chen J, et al. Plasma miRNAs as early biomarkers for detecting hepatocellular carcinoma. *International journal of cancer.* 2015.
9. Zhu C, Ren C, Han J, et al. A five-microRNA panel in plasma was identified as potential biomarker for early detection of gastric cancer. *British journal of cancer.* 2014;110(9):2291-2299.
10. Du M, Liu S, Gu D, et al. Clinical potential role of circulating microRNAs in early diagnosis of colorectal cancer patients. *Carcinogenesis.* 2014;35(12):2723-2730.
11. Zheng G, Du L, Yang X, et al. Serum microRNA panel as biomarkers for early diagnosis of colorectal adenocarcinoma. *British journal of cancer.* 2014;111(10):1985-1992.
12. Wang J, Huang SK, Zhao M, et al. Identification of a circulating microRNA signature for colorectal cancer detection. *PloS one.* 2014;9(4):e87451.
13. Liu GH, Zhou ZG, Chen R, et al. Serum miR-21 and miR-92a as biomarkers in the diagnosis and prognosis of colorectal cancer. *Tumour biology : the journal of the International Society for Oncodevelopmental Biology and Medicine.* 2013;34(4):2175-2181.
14. Wang S, Wang L, Bayaxi N, et al. A microRNA panel to discriminate carcinomas from high-grade intraepithelial neoplasms in colonoscopy biopsy tissue. *Gut.* 2013;62(2):280-289.
15. Luo X, Stock C, Burwinkel B, Brenner H. Identification and evaluation of plasma microRNAs for early detection of colorectal cancer. *PloS one.* 2013;8(5):e62880.
16. Torres A, Torres K, Pesci A, et al. Diagnostic and prognostic significance of miRNA signatures in tissues and plasma of endometrioid endometrial carcinoma patients. *International journal of cancer.* 2013;132(7):1633-1645.
17. Wu CW, Ng SS, Dong YJ, et al. Detection of miR-92a and miR-21 in stool samples as potential screening biomarkers for colorectal cancer and polyps. *Gut.* 2012;61(5):739-745.
18. Wang Q, Huang Z, Ni S, et al. Plasma miR-601 and miR-760 Are Novel Biomarkers for the Early Detection of Colorectal Cancer. *PloS one.* 2012;7(9).
19. Baraniskin A, Kuhnhenn J, Schlegel U, et al. Identification of microRNAs in the cerebrospinal fluid as marker for primary diffuse large B-cell lymphoma of the central nervous system. *Blood.* 2011;117(11):3140-3146.
20. Ng EKO, Chong WWS, Jin H, et al. Differential expression of microRNAs in plasma of patients with colorectal cancer: A potential marker for colorectal cancer screening. *Gut.* 2009;58(10):1375-1381.
21. Huang Z, Huang D, Ni S, Peng Z, Sheng W, Du X. Plasma microRNAs are promising novel biomarkers for early detection of colorectal cancer. *International journal of cancer.* 2010;127(1):118-126.
22. Li F, Guo Y, Liu J, Zhang R. The significance of elevated plasma expression of microRNA 106b~25 clusters in gastric cancer. *PloS one.* 2017;12(5).
23. Le Q, Jianhua N, Mulati, Yu X, Jiageng H. Increased miR-25 expression in serum of gastric

cancer patients is correlated with CA19-9 and acts as a potential diagnostic biomarker. *Open Medicine (Poland).* 2017;12(1):266-270.

24. Fujiwara T, Uotani K, Yoshida A, et al. Clinical significance of circulating miR-25-3p as a novel diagnostic and prognostic biomarker in osteosarcoma. *Oncotarget.* 2017;8(20):33375-33392.
25. Johansen JS, Calatayud D, Albieri V, et al. The potential diagnostic value of serum microRNA signature in patients with pancreatic cancer. *International journal of cancer.* 2016;139(10):2312-2324.
26. Deng T, Yuan Y, Zhang C, et al. Identification of Circulating miR-25 as a Potential Biomarker for Pancreatic Cancer Diagnosis. *Cellular Physiology and Biochemistry.* 2016;39(5):1716-1722.
27. Cochetti G, Poli G, Guelfi G, Boni A, Egidi MG, Mearini E. Different levels of serum microRNAs in prostate cancer and benign prostatic hyperplasia: Evaluation of potential diagnostic and prognostic role. *OncoTargets and Therapy.* 2016;9:7545-7553.
28. Cappellesso R, Nicolè L, Caroccia B, et al. Young investigator challenge: MicroRNA-21/MicroRNA-126 profiling as a novel tool for the diagnosis of malignant mesothelioma in pleural effusion cytology. *Cancer cytopathology.* 2016;124(1):28-37.
29. Bryzgunova OE, Zaripov MM, Skvortsova TE, et al. Comparative study of extracellular vesicles from the urine of healthy individuals and prostate cancer patients. *PloS one.* 2016;11(6).
30. Butz H, Nofech-Mozes R, Ding Q, et al. Exosomal MicroRNAs Are Diagnostic Biomarkers and Can Mediate Cell-Cell Communication in Renal Cell Carcinoma. *European Urology Focus.* 2016;2(2):210-218.
31. Zhou X, Zhu W, Li H, et al. Diagnostic value of a plasma microRNA signature in gastric cancer: a microRNA expression analysis. *Scientific reports.* 2015;5:11251.
32. Wang C, Wang X, Su Z, Fei H, Liu X, Pan Q. miR-25 promotes hepatocellular carcinoma cell growth, migration and invasion by inhibiting RhoGDI1. *Oncotarget.* 2015;6(34):36231-36244.
33. Meng X, Joosse SA, Müller V, et al. Diagnostic and prognostic potential of serum MIR-7, MIR-16, MIR-25, MIR-93, MIR-182, MIR-376a and MIR-429 in ovarian cancer patients. *British journal of cancer.* 2015;113(9):1358-1366.
34. Li M, Song Q, Li H, Lou Y, Wang L. Erratum: Circulating MIR-25-3p and MIR-451a may be potential biomarkers for the diagnosis of papillary thyroid carcinoma (PLoS ONE (2015) 10:7 (e0132403) DOI:10.1371/journal.pone.0132403). *PloS one.* 2015;10(8).
35. Jia W, Wu Y, Zhang Q, Gao G, Zhang C, Xiang Y. Expression profile of circulating microRNAs as a promising fingerprint for cervical cancer diagnosis and monitoring. *Molecular and Clinical Oncology.* 2015;3(4):851-858.
36. Wang P, Yang D, Zhang H, et al. Early detection of lung cancer in serum by a panel of MicroRNA biomarkers. *Clinical Lung Cancer.* 2015;16(4):313-319.e311.
37. Wu C, Wang C, Guan X, et al. Diagnostic and prognostic implications of a serum miRNA panel in oesophageal squamous cell carcinoma. *PloS one.* 2014;9(3):e92292.
38. Pan W, Tang W, Yuan W, et al. Expression and clinical significance of plasma small RNA in patients with pancreatic cancer. *Chinese Journal of Oncology.* 2014;36(5):351-354.
39. Mengual L, Lozano JJ, Ingelmo-Torres M, Gazquez C, Ribal MJ, Alcaraz A. Using microRNA profiling in urine samples to develop a non-invasive test for bladder cancer. *International journal of cancer.* 2013;133(11):2631-2641.
40. Hu Z, Dong J, Wang LE, et al. Serum microRNA profiling and breast cancer risk: The use of miR-484/191 as endogenous controls. *Carcinogenesis.* 2012;33(4):828-834.

41. Li LM, Hu ZB, Zhou ZX, et al. Serum microRNA profiles serve as novel biomarkers for HBV infection and diagnosis of HBV-positive hepatocarcinoma. *Cancer Research.* 2010;70(23):9798-9807.
42. Wu CW, Cao X, Berger CK, et al. Novel Approach to Fecal Occult Blood Testing by Assay of Erythrocyte-Specific microRNA Markers. *Digestive Diseases and Sciences.* 2017;62(8):1985-1994.
43. Ingelmo-Torres M, Lozano JJ, Izquierdo L, et al. Urinary cell microRNA-based prognostic classifier for nonmuscle invasive bladder cancer. *Oncotarget.* 2017;8(11):18238-18247.
44. Ren C, Wang W, Han C, et al. Expression and prognostic value of miR-92a in patients with gastric cancer. *Tumour biology : the journal of the International Society for Oncodevelopmental Biology and Medicine.* 2016;37(7):9483-9491.
45. Yang W, Dou C, Wang Y, et al. MicroRNA-92a contributes to tumor growth of human hepatocellular carcinoma by targeting FBXW7. *Oncol Rep.* 2015;34(5):2576-2584.
46. Zhou T, Zhang G, Liu Z, Xia S, Tian H. Overexpression of miR-92a correlates with tumor metastasis and poor prognosis in patients with colorectal cancer. *International journal of colorectal disease.* 2013;28(1):19-24.
47. Nilsson S, Moller C, Jirstrom K, et al. Downregulation of miR-92a is associated with aggressive breast cancer features and increased tumour macrophage infiltration. *PloS one.* 2012;7(4):e36051.
48. Jacob H, Stanisavljevic L, Storli KE, Hestetun KE, Dahl O, Myklebust MP. Identification of a sixteen-microRNA signature as prognostic biomarker for stage II and III colon cancer. *Oncotarget.* 2017;8(50):87837-87847.
49. Goossens-Beumer IJ, Derr RS, Buermans HPJ, et al. MicroRNA classifier and nomogramfor metastasis prediction in colon cancer. *Cancer Epidemiology Biomarkers and Prevention.* 2015;24(1):187-197.
50. Wu C, Li M, Hu C, Duan H. Clinical significance of serum miR-223, miR-25 and miR-375 in patients with esophageal squamous cell carcinoma. *Mol Biol Rep.* 2014;41(3):1257-1266.
51. Wang X, Meng X, Li H, Liu W, Shen S, Gao Z. MicroRNA-25 expression level is an independent prognostic factor in epithelial ovarian cancer. *Clinical and Translational Oncology.* 2014;16(11):954-958.
52. Su ZX, Zhao J, Rong ZH, Geng WM, Wu YG, Qin CK. Upregulation of microRNA-25 associates with prognosis in hepatocellular carcinoma. *Diagnostic pathology.* 2014;9:47.
53. Li X, Yang C, Wang X, Zhang J, Zhang R, Liu R. The expression of miR-25 is increased in colorectal cancer and is associated with patient prognosis. *Medical Oncology.* 2014;31(1).
54. Xu FX, Su YL, Zhang H, Kong JY, Yu H, Qian BY. Prognostic implications for high expression of MiR-25 in lung adenocarcinomas of female non-smokers. *Asian Pacific Journal of Cancer Prevention.* 2014;15(3):1197-1203.
55. Jonsdottir K, Janssen SR, Da Rosa FC, et al. Validation of Expression Patterns for Nine miRNAs in 204 Lymph-Node Negative Breast Cancers. *PloS one.* 2012;7(11).
